# Plasma cytokine profiling identifies early kidney injury and systemic inflammation in prurigo nodularis

**DOI:** 10.1016/j.jdin.2025.08.002

**Published:** 2025-08-19

**Authors:** Yagiz Matthew Akiska, Sara Khoshniyati, Deena Fayyad, Shabnam Afzal, Perya Bhagchandani, Davies Gage, Shahin Shahsavari, Louis J. Born, Kavita Vats, Shivani S. Patel, Shawn G. Kwatra

**Affiliations:** a Department of Dermatology, University of Maryland School of Medicine, Baltimore, Maryland; b Maryland Itch Center, University of Maryland School of Medicine, Baltimore, Maryland

**Keywords:** cytokine profiling, precision medicine, prurigo nodularis, renal injury, systemic inflammation

*To the Editor:* Prurigo nodularis (PN) is a chronic skin disorder characterized by intensely pruritic, hyperkeratotic nodules that impair quality of life.^1^ Traditionally considered a condition confined to the skin, PN is increasingly recognized as a systemic inflammatory disease.^1^ Recent epidemiologic studies highlight a strong association between PN and kidney dysfunction, with a 1.5-fold increased risk of chronic kidney disease (CKD) and end-stage renal disease.^2^ However, the biological mechanisms underlying this association remain unclear. To address this gap, we conducted a cross-sectional study assessing circulating biomarkers of kidney injury and systemic inflammation in patients with PN versus healthy controls (HCs). All PN patients had dermatologist-confirmed diagnoses, ≥ 20 nodules, and a Worst Itch Numeric Rating Scale ≥ 7.

Plasma samples from 20 PN patients and 9 matched HCs were analyzed using multiplex Luminex assays to quantify kidney and cardiac injury–associated cytokines. Patients with known kidney or cardiac comorbidities, including CKD, heart disease, hypertension, and chronic obstructive pulmonary disease, were excluded to minimize confounding. Linear and logistic regression analyses were conducted, adjusting for age, sex, and race.

The PN cohort (*n* = 20) had a median age of 51.0 years (interquartile range: 44.0-63.3) compared to 34.0 years (interquartile range: 28.0-52.0) in the HC cohort (*n* = 9), although this difference was not statistically significant (*P* = .085). Both cohorts had similar sex distributions (PN: 85.0% female; HC: 55.6% female; *P* = .158) and racial distributions (*P* = .431). PN patients exhibited significantly elevated levels of multiple kidney injury–associated biomarkers compared to HCs, including α1-microglobulin, collagen IV, lipocalin-2, osteoactivin, Tissue Inhibitor of Metalloproteinase-1, and uromodulin (all *P* < .001) (Table I). Of these, osteoactivin/glycoprotein nonmetastatic melanoma protein B/dendritic cell heparin integrin ligand demonstrated the strongest association with PN status (odds ratio = 4.71; 95% confidence interval: 1.86-11.97) (Fig 1). For cardiac biomarkers, L-selectin (Δ = −110,215.05 ng/mL; *P* = .03) and platelet factor 4 (Δ = −343.77 ng/mL; *P* = .03) were significantly reduced in PN compared to HCs, with no significant differences in other markers (all *P* > .05).

**Table I:** Patient demographics and multivariate linear regression analysis of kidney and cardiac-associated cytokines in PN vs healthy controls

Demographics	HC (*n* = 9)	PN (*n* = 20)	*P* value
Median age (IQR)	34.0 (28.0-52.0)	51.0 (44.0-63.25)	.0851
Race			.431
Caucasian	7 (77.8)	12 (60.0)	
African American	2 (22.2)	8 (40.0)	
Sex (%)			.158
Female	5 (55.6)	17 (85.0)	
Male	4 (44.4)	3 (15.0)	
WI-NRS (SD)	0 (0)	8.30 (2.13)	<.0001
Disease severity score (IGA)	0	3.32 ± 0.67	

Cytokine	Effect size∗	95% CI	*P* value (adjusted)∗
Osteoactivin/GPNMB/DC-HIL	1.69	[1.11, 2.27]	.0000263
Collagen IV	1.55	[1.02, 2.09]	.0000263
α1-microglobulin	1.67	[1.07, 2.27]	.0000302
Uromodulin	1.59	[0.93, 2.25]	.000171
TIMP-1	1.40	[0.74, 2.06]	.000635
Lipocalin-2	1.42	[0.65, 2.19]	.00217
α2-macroglobulin	−0.57	[−1.10, −0.04]	.0809
SAP	0.61	[−0.01, 1.23]	.0921
Fetuin A36	−0.18	[−0.36, 0.00]	.0921
L-selectin	−0.48	[−0.98, 0.02]	.0921
CRP	0.89	[−0.09, 1.88]	.0997
Haptoglobulin	0.85	[−0.12, 1.82]	.103
PF4	−0.67	[−1.45, 0.11]	.103
AGP	0.23	[−0.35, 0.82]	.450
Fibrinogen	−0.07	[−0.52, 0.38]	.739

*AGP*, Alpha-1-acid glycoprotein; *CI*, confidence interval; *CRP*, C-reactive protein; *DC-HIL*, dendritic cell heparin integrin ligand; *GPNMB*, glycoprotein nonmetastatic melanoma protein B; *HC*, healthy control; *IGA*, Investigator’s Global Assessment; *IQR*, interquartile range; *PF4*, platelet factor 4; *PN*, prurigo nodularis; *SAP*, serum amyloid P; *SD*, standard deviation; *TIMP-1*, Tissue Inhibitor of Metalloproteinase-1; *WI-NRS*, Worst Itch Numeric Rating Scale.

∗ Effect sizes and 95% CIs from linear regression models relating PN status to standardized (z-score) cytokine levels, controlling for age, sex, and race. Positive effect sizes indicate higher mean cytokine levels in PN compared to healthy controls. *P* values were adjusted for multiple comparisons using the Benjamini-Hochberg method.

**Fig 1:**
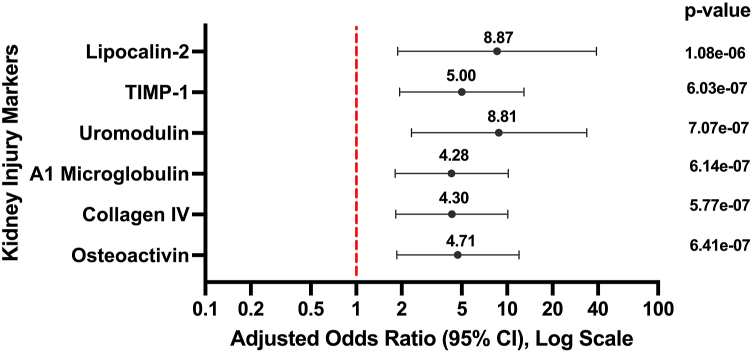
Forest plot of adjusted odds ratios for kidney injury biomarkers associated with PN. Forest plot illustrates the adjusted odds ratios (aORs), 95% confidence intervals (CIs), and *P* values from logistic regression models for a 1-unit increase in log-transformed cytokine levels, adjusted for age. An OR more than 1 indicates higher odds of PN. The dashed *vertical line* at OR = 1 represents the null value. *95% CI*, 95% Confidence interval; *PN*, prurigo nodularis.

Our study reveals a distinct cytokine profile in PN, with elevated kidney injury markers suggesting systemic inflammation and subclinical renal stress. Notably, osteoactivin may be a key mediator of PN pathophysiology through its roles in fibrotic signaling^3^ and eosinophil development.^4^ Given that patients with advanced kidney disease frequently experience pruritus, it is plausible that PN and CKD may share a bidirectional relationship. Although we found no significant differences in traditional cardiac biomarkers, elevated tissue remodeling markers like collagen IV suggest vascular stress. Modest reductions in L-selectin and platelet factor 4 may reflect altered leukocyte trafficking or platelet activity, warranting further study.^5^

These insights reinforce the evolving perspective of PN as a systemic disorder and support a more comprehensive approach to disease management. Routine assessment of renal stress markers could provide an opportunity for early intervention before irreversible kidney damage occurs. Additionally, early systemic treatment and lifestyle modifications could mitigate metabolic and renal stress. These interventions may ultimately improve outcomes and reduce the burden of comorbid disease.

Limitations of our study include a small sample size and cross-sectional design. Longitudinal studies are needed to further assess the relationship between PN and renal dysfunction.

## Conflicts of interest

Dr Shawn G. Kwatra is an advisory board member/consultant for AbbVie, Amgen, Arcutis Biotherapeutics, Aslan Pharmaceuticals, Cara Therapeutics, Castle Biosciences, Celldex Therapeutics, Galderma, Genzada Pharmaceuticals, Incyte Corporation, Johnson & Johnson, Leo Pharma, Novartis Pharmaceuticals Corporation, Pfizer, Regeneron Pharmaceuticals, and Sanofi and has served as an investigator for Galderma, Incyte, Pfizer, and Sanofi. All other authors report no conflicts of interest.
